# Supplementary material for: A Facile Method for the Removal of dsRNA Contaminant from In Vitro-Transcribed mRNA
Source: Mol Ther Nucleic Acids. 2019 Feb 27;15:26–35. doi: 10.1016/j.omtn.2019.02.018 (PMC6444222; doi:10.1016/j.omtn.2019.02.018)
Supplement: Document S1. Figures S1 and S2 [file mmc1.pdf]

OMTN, Volume *15*

Supplemental Information

A Facile Method for the Removal of dsRNA Contaminant from *In Vitro*-Transcribed mRNA

Markus Baiersdörfer, Gábor Boros, Hiromi Muramatsu, Azita Mahiny, Irena Vlatkovic, Ugur Sahin, and Katalin Karikó

Supplementary Fig. 1

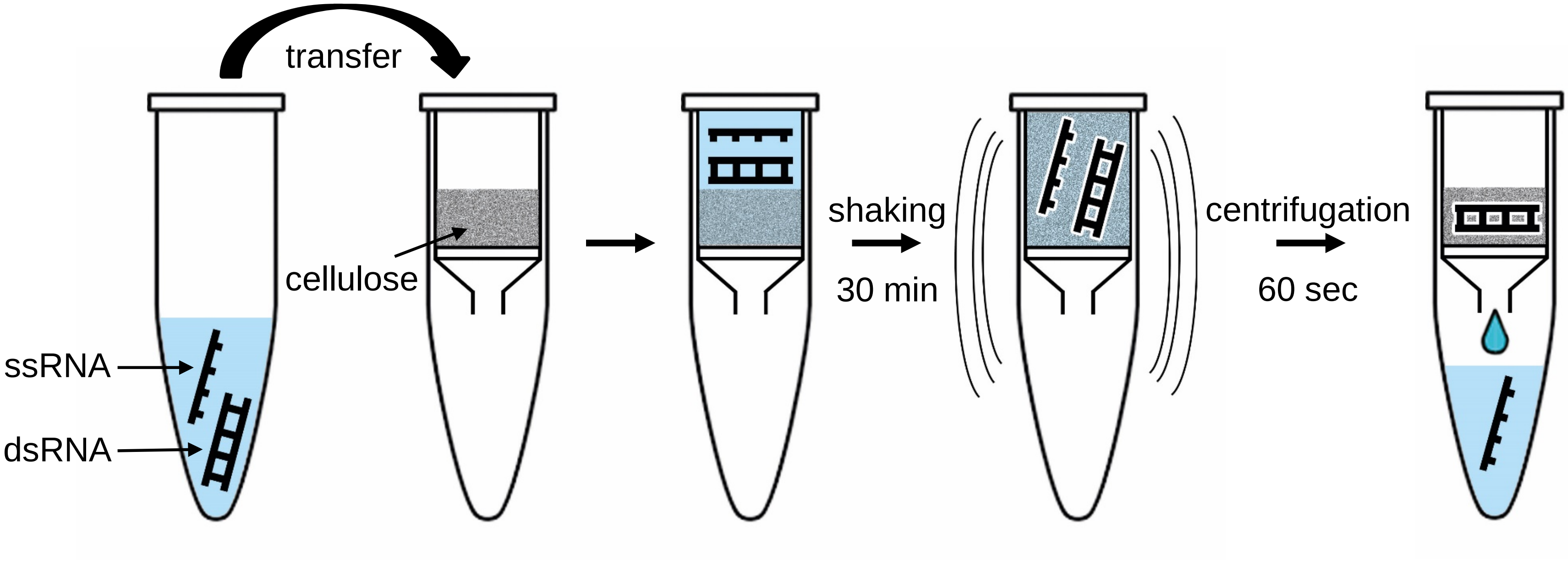

Supplementary Fig. 2

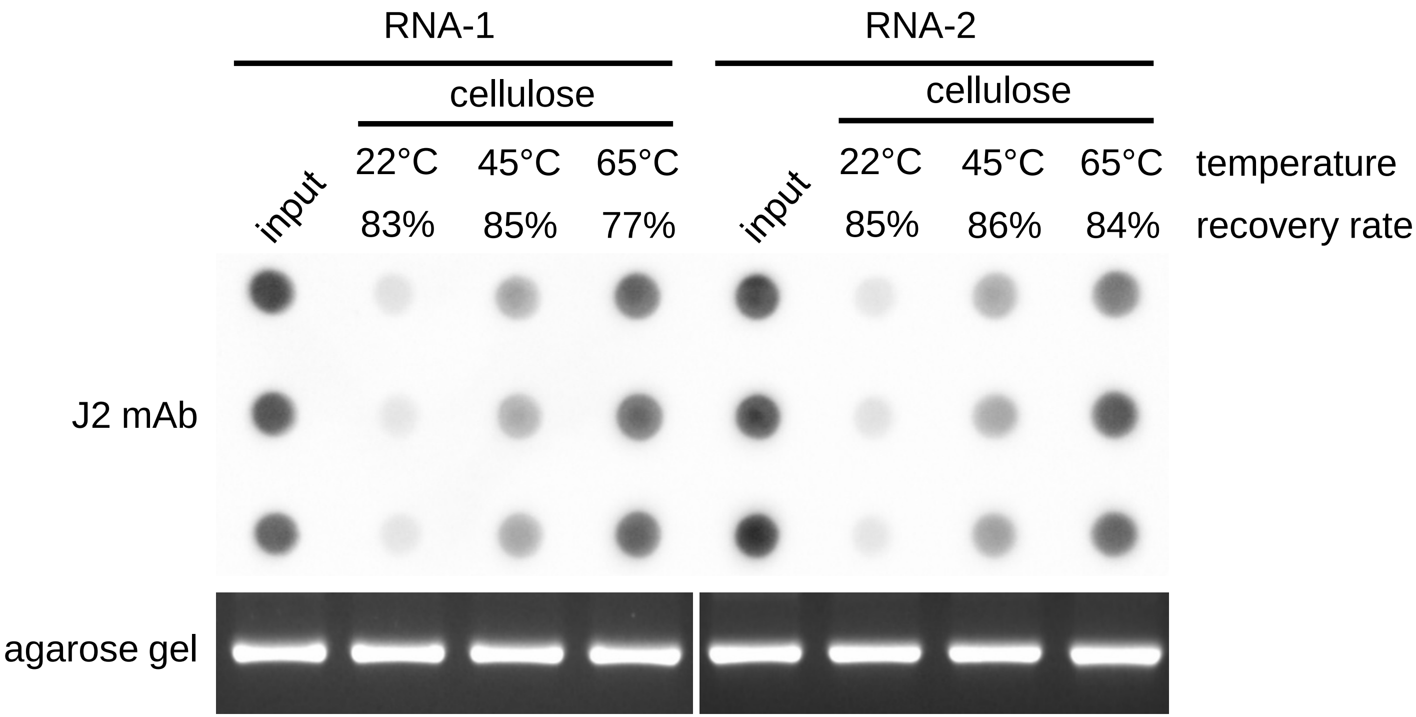

SUPPLEMENTARY MATERIALS

Supplementary Figure 1. Schematic overview of the cellulose-based purification method using microcentrifuge spin column. IVT mRNA in chromatography buffer containing 16% (v/v) ethanol is transferred to cellulose-loaded microcentrifuge spin column. The spin column is shaken vigorously at room temperature for 30 minutes to promote resuspension of the cellulose and its association with the dsRNA contaminant contained in the IVT mRNA. By centrifugation of the spin column at 14,000 x g for 60 seconds the cellulose together with the associated dsRNA contaminant is separated from the unbound single-stranded IVT mRNA (ssRNA) in the flow through.

Supplementary Figure 2. High temperatures decrease the efficiency of dsRNA removal with cellulose chromatography. Two different uridine-containing, 1.9 kb-long IVT mRNAs (RNA-1 and RNA-2) were purified at 22°C, 45°C or 65°C using cellulose-filled microcentrifuge spin columns. Aliquots of the unpurified (input) and cellulose-purified IVT mRNAs (1 µg per dot) were analyzed in triplicates on dot blot with J2 dsRNA-specific mAb for presence of dsRNA contaminants. The recovery rates of the purified mRNAs relative to the input mRNA are noted. The integrities of unpurified and cellulose-purified IVT mRNAs were analyzed by electrophoresis on a 1.4% agarose gel. The GelRed-stained RNAs were visualized by UV illumination.
