# Supplementary material for: A Facile Method for the Removal of dsRNA Contaminant from In Vitro-Transcribed mRNA
Source: Mol Ther Nucleic Acids. 2019 Feb 27;15:26–35. doi: 10.1016/j.omtn.2019.02.018 (PMC6444222; doi:10.1016/j.omtn.2019.02.018)
Supplement: Document S2. Article plus Supplemental Information [file mmc2.pdf]

Molecular Therapy
Nucleic Acids
Original Article

AMERICAN SOCIETY of GENE & CELL THERAPY

A Facile Method for the Removal of dsRNA Contaminant from *In Vitro*-Transcribed mRNA

Markus Baiersdörfer,[1] Gábor Boros,[1] Hiromi Muramatsu,[1,2] Azita Mahiny,[1] Irena Vlatkovic,[1] Ugur Sahin,[1] and Katalin Karikó[1]

[1]BioNTech RNA Pharmaceuticals, 55131 Mainz, Germany

The increasing importance of *in vitro*-transcribed (IVT) mRNA for synthesizing the encoded therapeutic protein *in vivo* demands the manufacturing of pure mRNA products. The major contaminant in the IVT mRNA is double-stranded RNA (dsRNA), a transcriptional by-product that can be removed only by burdensome procedure requiring special instrumentation and generating hazardous waste. Here we present an alternative simple, fast, and cost-effective method involving only standard laboratory techniques. The purification of IVT mRNA is based on the selective binding of dsRNA to cellulose in an ethanol-containing buffer. We demonstrate that at least 90% of the dsRNA contaminants can be removed with a good, >65% recovery rate, regardless of the length, coding sequence, and nucleoside composition of the IVT mRNA. The procedure is scalable; purification of microgram or milligram amounts of IVT mRNA is achievable. Evaluating the impact of the mRNA purification *in vivo* in mice, increased translation could be measured for the administered transcripts, including the 1-methylpseudouridine-containing IVT mRNA, which no longer induced interferon (IFN)-α. The cellulose-based removal of dsRNA contaminants is an effective, reliable, and safe method to obtain highly pure IVT mRNA suitable for *in vivo* applications.

INTRODUCTION

In recent years, *in vitro*-transcribed (IVT) mRNA for research and therapeutic applications has gained tremendous interest (reviewed by Sahin et al.[1]). The simplicity of the approach to synthesize almost any protein *in vitro* or *in vivo* by delivering the encoding IVT mRNA makes it very appealing. Phage RNA polymerases, including T7 RNA polymerase (T7RNAPol), transcribe the RNA with high fidelity from a DNA template containing the corresponding promoter.[2] However, during the initiation of transcription, 5- to 11-nt-long RNA by-products are generated as the enzyme aborts the synthesis with a certain probability.[3,4] Considering that the T7RNAPol also has an obscure RNA-dependent as well as template-independent RNA polymerase activity,[5–12] the short abortive RNA fragments and the 3′ end of the full-length RNA can prime complementary RNA synthesis from the primary transcripts that leads to the generation of double-stranded RNA (dsRNA) contaminant. Recently, a promoter-independent transcription of full-length anti-sense RNA has been also reported as a novel mechanism of dsRNA generation in T7RNAPol-driven IVT reaction.[13]

The nuclei of human cells contain dsRNA that plays a role in natural biological processes;[14,15] however, when dsRNA enters the cells, from the extracellular milieu into their endosome or cytoplasm, it is sensed as a viral invader. All cells are capable of responding to dsRNA through sensors and effectors. Activation of dsRNA-dependent enzymes, such as oligoadenylate synthetase (OAS), RNA-specific adenosine deaminase (ADAR), and RNA-activated protein kinase (PKR), results in the inhibition of protein synthesis. In addition, stimulation of the dsRNA sensors, e.g., Toll-like receptor 3 (TLR3), retinoic acid-inducible gene I (RIG-I), and melanoma differentiation-associated protein 5 (MDA5), leads to the secretion of different cytokines, including type I interferons, interleukin-6 (IL-6), and tumor necrosis factor-α (TNF-α) (reviewed by Hartmann[16]). Therefore, the elimination of dsRNA from the IVT mRNA is crucial to improve translation of the administered mRNA and to limit induction of cytokines, whether the mRNA products are intended for *in vitro*, *ex vivo*, or *in vivo* applications and to be delivered by electroporation or in formulation.

The dsRNA impurities are not efficiently removed from IVT mRNA during standard purification methods, including LiCl or alcohol-based precipitation, size exclusion and ion exchange chromatography, or purification based on silica matrices. To date the most effective way to eliminate dsRNA contaminants from long IVT mRNAs is using ion pair reversed-phase high-performance liquid chromatography (HPLC).[17,18] However, this method is not scalable, the acetonitrile eluent is very toxic, and, most importantly, it is unaffordable for most laboratories.

Received 23 October 2018; accepted 19 February 2019;
https://doi.org/10.1016/j.omtn.2019.02.018.

[2]Present address: Department of Medicine, University of Pennsylvania, Philadelphia, PA 19104, USA

Correspondence: Markus Baiersdörfer, BioNTech RNA Pharmaceuticals, An der Goldgrube 12, Rheinland-Pfalz, 55131 Mainz, Germany.
E-mail: markus.baiersdoerfer@biontech.de

 © 2019 The Author(s).
This is an open access article under the CC BY license (http://creativecommons.org/licenses/by/4.0/).

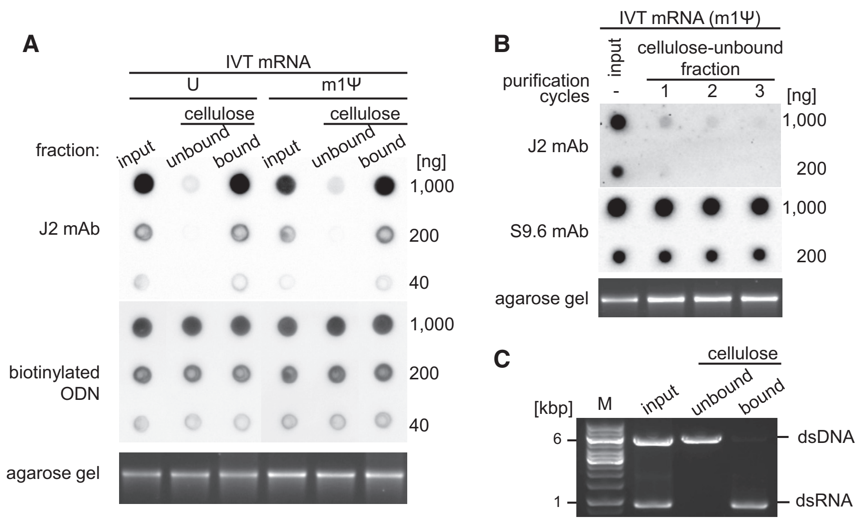

Figure 1. Evaluating the Purity of IVT mRNA following Cellulose Chromatography

(A) Uridine (U)- or 1-methylpseudouridine (m1Ψ)-containing, 2.2-kb-long IVT mRNA present in 16% ethanol-containing buffer was purified using cellulose-filled microcentrifuge spin columns. A dot blot loaded with aliquots of mRNA was analyzed for the presence of dsRNA contaminants with J2 mAb and reprobed with complementary oligodeoxynucleotide (ODN). The corresponding mRNA fractions were analyzed by electrophoresis on a 1.4% agarose gel. The GelRed-stained RNAs were visualized by UV illumination. (B) *In vitro*-transcribed mRNA containing m1Ψ was purified repeatedly in three consecutive cycles using cellulose-filled microcentrifuge spin columns. Aliquots of mRNA were analyzed in dot blot with J2 dsRNA-specific mAb and S9.6 mAb specific for RNA:DNA hybrids. The mRNA fractions separated in 1.4% agarose gel were stained with GelRed and visualized by UV illumination. (C) Linearized plasmid and U-containing dsRNA were mixed in 16% ethanol-containing buffer and then subjected to cellulose chromatography. DNA recovered from the unbound fraction and dsRNA eluted from the cellulose-bound fraction were separated on 1% agarose gel, then stained with GelRed and visualized by UV illumination. M, DNA molecular weight marker.

Here we present a simple, scalable, and safe method for the removal of dsRNA contaminants from IVT mRNA, requiring only standard laboratory procedures. The principle of separation involves the selective binding of dsRNA to conventional cellulose powder in ethanol-containing buffer. This IVT mRNA purification procedure was adapted from a method established for the isolation of viral and fungal dsRNA from plant tissues.[19–21] The cellulose-based isolation of dsRNA has been used for decades and undergone only minor modifications during the years, e.g., using commercial mini-columns packed with cellulose powders, to speed up the process and to increase the sample throughput.[22–24] We tested the feasibility of this cellulose-based separation for removing dsRNA contaminants from IVT mRNA to obtain dsRNA-free, pure mRNA product.

RESULTS

dsRNA Contaminants in IVT mRNAs Bind to Cellulose in the Presence of Ethanol

To test the feasibility of applying cellulose to remove dsRNA contaminants from IVT RNA, first a simple separation experiment was performed (Figure S1). Purification of the uridine (U)- and 1-methylpseudouridine (m1Ψ)-containing 2-kb-long IVT mRNA encoding luciferase (Luc) was performed in 16% ethanol-containing buffer using microcentrifuge spin columns filled with cellulose. From the flowthrough fraction, 70%–80% of the input IVT mRNA could be recovered as unbound mRNA. The dot blot analysis performed with J2 dsRNA-specific antibody demonstrated that the fraction of unbound mRNA contained approximately 90% less dsRNA compared to the corresponding input mRNA, thus confirming the efficient removal of dsRNA contaminants by cellulose (Figure 1A). In contrast, the cellulose-bound mRNA fractions that were eluted with nuclease-free water after the purification step contained dsRNA in amounts slightly higher than the corresponding input IVT mRNA. This confirms the association of dsRNA with the cellulose material during the purification process performed at room temperature. When the purifications were conducted at higher temperature (e.g., 45°C and 65°C), the recovery rates of RNA were unchanged, but significantly more dsRNA contaminants remained in the unbound fractions (Figure S2). Analysis of the collected mRNA fractions by agarose gel electrophoresis demonstrated that their integrity was not compromised during incubation with the cellulose material (Figure 1A). Binding of double-stranded nucleic acids to cellulose in the 16% ethanol-containing buffer is a unique property of dsRNA, as RNA:DNA hybrid or double-stranded DNA (dsDNA) remain in the unbound fractions under these conditions (Figures 1B and 1C).

To confirm that dsRNA can be removed from IVT mRNA with cellulose and to estimate the dsRNA-binding capacity of cellulose, we generated a 1-kb-long m1Ψ-containing dsRNA by annealing of two complementary IVT RNAs, and we spiked it into m1Ψ-containing 1-kb-long IVT mRNA to a final concentration of 0.02–20 ng dsRNA/μg IVT mRNA. After purification using cellulose-filled spin columns, the mRNA was recovered from the flowthrough, unbound fraction. Dot blot analysis showed a strong reduction of dsRNA-specific signal in all mRNA samples that had been purified by cellulose chromatography (Figure 2A). The input mRNA samples, as expected, led to increasing J2 antibody signal intensities that corresponded with the amount of spiked dsRNA.

Notably, the IVT-derived dsRNA contaminants as well as the spiked dsRNA were removed, indicating that the dsRNA-binding capacity of the cellulose material is higher than the total amount of dsRNA

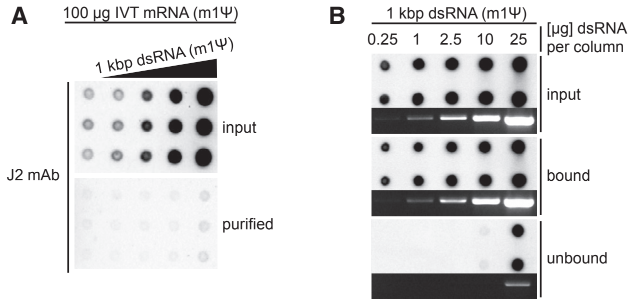

Figure 2. Removal of Long dsRNA from IVT mRNA by Cellulose Chromatography

(A) Purified, m1Ψ-containing, 1.0-kb-long mRNA was spiked with a 1-kb-long m1Ψ-containing dsRNA at final concentrations of 0.02–20 ng dsRNA/µg mRNA. 100 µg dsRNA-spiked mRNA sample was purified using microcentrifuge spin columns filled with 0.14 g cellulose. The purified mRNA recovered from the flowthrough, unbound fraction and the unpurified input mRNAs (1.0 µg/dot) were analyzed on dot blot. (B) The indicated amounts of 1 kb dsRNA containing m1Ψ were subjected to cellulose purification using microcentrifuge spin columns filled with 0.14 g cellulose. Aliquots of the dsRNA bound to the cellulose or recovered from the flowthrough fractions (unbound) and the input dsRNA were analyzed on dot blots using J2 dsRNA-specific mAb. Aliquots of the corresponding dsRNA samples were also analyzed in non-denaturing 1.4% agarose gel. The GelRed-stained samples were visualized by UV illumination.

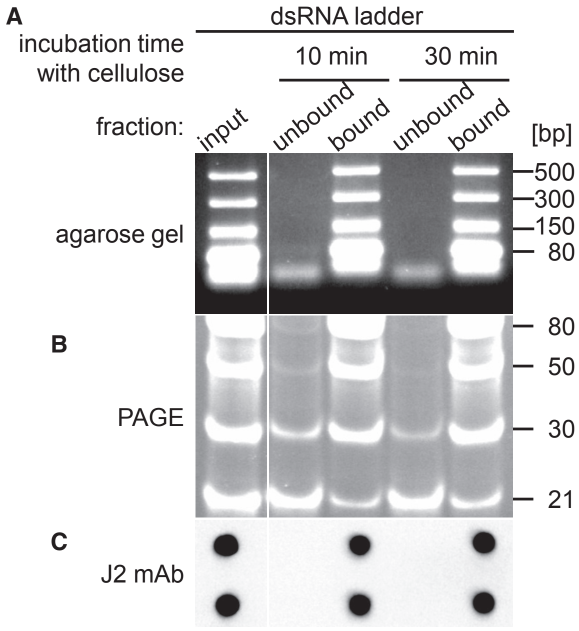

Figure 3. Cellulose Chromatography of dsRNA Ladder

Broad-range dsRNA ladder (21–500 bp), incubated for 10 or 30 min with cellulose in the presence of chromatography buffer containing 16% ethanol, was purified using microcentrifuge spin columns. The input dsRNA ladder and the unbound and the cellulose-bound fractions were analyzed by electrophoresis in (A) a non-denaturing 2.2% agarose gel, (B) a native 21% polyacrylamide gel (PAGE), and (C) by dot blot analysis using J2 dsRNA-specific mAb.

present in the input RNA and not a limiting factor in this experiment. To determine the maximal amount of dsRNA that can be removed with the purification protocol, we evaluated binding of increasing amounts of 1-kb-long dsRNA to the cellulose. In the spin column, 0.14 g cellulose bound nearly all the 10 µg m1Ψ-containing dsRNA, while 25 µg dsRNA clearly exceeded the binding capacity of the cellulose (Figure 2B). A similar result was obtained using U-containing dsRNA (data not shown).

Cellulose Chromatography Removes dsRNA Longer Than 30 bp

The dsRNAs generated during *in vitro* transcription are likely to have different lengths; thus, we tested the capacity of cellulose chromatography to remove dsRNAs ranging between 21 and 500 bp. A commercial dsRNA ladder was incubated with cellulose in the presence of ethanol-containing chromatography buffer for 10 or 30 min. Analysis of the unbound and cellulose-bound fractions by agarose gel electrophoresis demonstrated that dsRNAs with a length ≥80 bp were effectively removed by cellulose chromatography even after 10 min of incubation (Figure 3A). Electrophoresis of the samples in a native polyacrylamide gel showed that dsRNAs longer than 30 bp were eliminated, while the majority of the 21-bp-long dsRNA was still present in the unbound fraction even after 30 min of incubation (Figure 3B). These findings indicate that contaminating dsRNA with a length ≥30 bp can be removed efficiently from IVT mRNA with cellulose chromatography. Notably the signal intensities on the dot blot correlated with the amount of dsRNA at the size of ≥50 bp (Figure 3C), which is in good agreement with an earlier report showing that a minimal length of 40 bp dsRNA is necessary to be detected by J2 antibody.[25]

Purification of IVT mRNAs with Different Lengths by Cellulose Chromatography

Next, we tested whether cellulose chromatography is suitable to remove dsRNA contaminants from IVT mRNA of different lengths. Two mRNAs, the 4.0-kb- and 1.3-kb-long IVT mRNAs encoding human inducible nitric oxide synthase (hiNOS) and EGFP, respectively, were each transcribed as U- and m1Ψ-containing mRNA and purified using spin columns filled with cellulose. Since repeated cycles of cellulose chromatography increased the purity of IVT mRNA (Figure 1B), we performed two cycles of consecutive purification for this experiment.

Independently from the length and nucleoside composition, the IVT-derived dsRNA contaminants could be removed by cellulose chromatography (Figure 4A). Densitometric quantitation of the J2 antibody signals from the dot blot analysis indicated that ≥90% of dsRNA contaminants were removed by cellulose chromatography. The recovery rate of all purified mRNAs ranged between 70% and 80%. Furthermore, analysis by agarose gel electrophoresis demonstrated the high integrity of the purified mRNAs, confirming that IVT mRNA even with a length of 4.0 kb remains intact during the purification process (Figure 4B). Interestingly, both the 1.3- and 4.0-kb unpurified IVT m1Ψ-mRNAs contained significantly less dsRNA contaminant than the corresponding U-containing RNA.

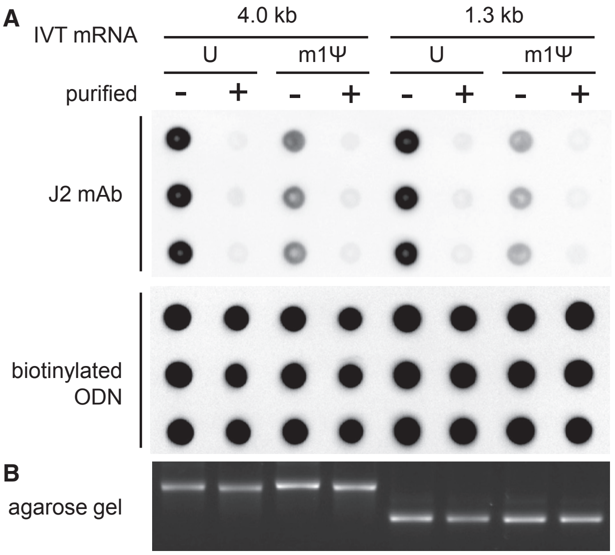

Figure 4. Purification of IVT mRNAs with Different Coding Sequences, Lengths, and Nucleoside Compositions Using Cellulose Chromatography
(A) U- and m1Ψ-containing hiNOS mRNA (4.0 kb) and EGFP mRNA (1.3 kb) were purified by two consecutive cycles using microcentrifuge spin columns filled with cellulose. Aliquots of the unpurified and purified IVT RNAs were analyzed in triplicates on dot blot with J2 dsRNA-specific mAb and with a complementary biotinylated oligodeoxynucleotide (ODN). (B) Aliquots of the IVT mRNAs were analyzed by electrophoresis on a 1.4% agarose gel.

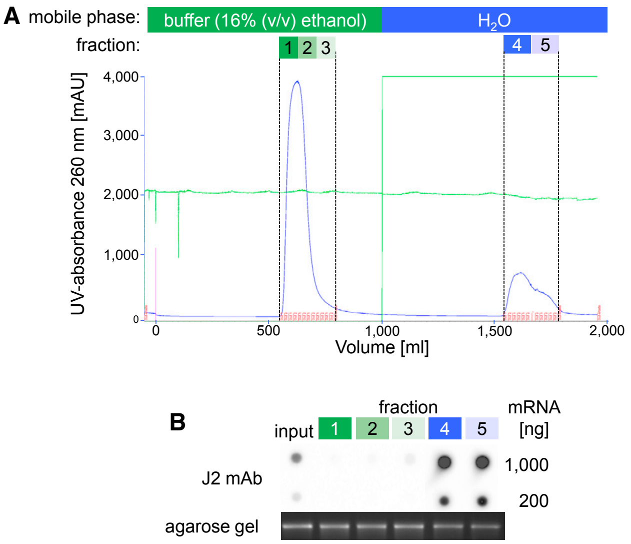

Figure 5. FPLC-Based Cellulose Chromatography of IVT mRNA
The total of 100 mg of a 2.2-kb-long U-containing IVT mRNA was purified by FPLC-based cellulose chromatography using chromatography buffer with 16% (v/v) ethanol, as described in the Materials and Methods. (A) The UV chromatogram of the FPLC purification run is shown. The collected fractions containing the eluted purified mRNA are marked as fractions 1–3, and the cellulose-bound RNA eluted with water is marked as fractions 4 and 5. (B) Analysis of the collected fractions by dot blot and electrophoresis on a 1.4% agarose gel.

The amount of mRNA that can be purified using microcentrifuge spin column is limited to about 500 μg RNA/column; thus, it was tested if the cellulose-based purification method can be transferred to a conventional fast protein liquid chromatography (FPLC) setting. This would allow a higher flexibility with respect to the amount of mRNA that can be purified in a single step. We performed an FPLC-based cellulose chromatography of 100 mg of a 2-kb U-containing Luc mRNA using a conventional glass column packed with 250 g cellulose. The UV-chromatogram shows the elution of the unbound mRNA as a single peak (Figure 5A, fractions 1–3), with chromatography buffer containing 16% ethanol as a mobile phase. The cellulose-bound mRNA was eluted after changing the mobile phase to water (Figure 5A, fractions 4 and 5).

The purified mRNA recovered from fractions 1–3 contained significantly less dsRNA than the input mRNA, demonstrating the successful purification of the mRNA by FPLC-based cellulose chromatography (Figure 5B). Densitometric analysis of the J2 antibody signal indicated that >95% of the dsRNA had been removed from the mRNA. The cellulose-bound mRNA (fractions 4 and 5) contained substantially higher concentrations of dsRNA compared to the input mRNA, demonstrating the enrichment of dsRNA contaminants in these fractions. The recovery rate of purified mRNA (fractions 1–3) was about 75%. The efficiency of purification was slightly better in fractions 1 and 2 than in fraction 3 of the main peak. However, since the mRNA amount in fraction 3 represented only about 5% of the total amount of purified mRNA, this had no impact on the overall purity. Most importantly, agarose gel electrophoresis showed that the integrity of the purified mRNA (fractions 1–3) and the dsRNA-enriched material (fractions 4 and 5) was identical to that of the unpurified input mRNA, indicating the absence of RNA degradation during the chromatography (Figure 5B). These results are representative of more than 30 independent FPLC runs using 5–100 mg mRNA and appropriately sized columns, and they demonstrate the feasibility of the cellulose chromatography for purifying large quantities of IVT mRNA.

Cellulose-Purified IVT mRNA Translates Better *In Vivo*

Elimination of dsRNA contaminants by HPLC has been shown to enhance translation of IVT mRNA.[17] To compare the translational capacity of IVT mRNA purified either with cellulose chromatography or HPLC, we generated both U- and m1Ψ-containing mRNAs encoding murine erythropoietin (mEPO), and we purified them by two consecutive cycles using microcentrifuge columns filled with cellulose or by HPLC. The mRNAs contained significantly less dsRNA contaminants after both cellulose chromatography and HPLC (Figure 6A). The J2 antibody signal intensities of the mRNAs purified by HPLC or cellulose chromatography were approximately equal, demonstrating that the efficiency of dsRNA removal by both purification methods was similar. Accordingly, the plasma of animals at 6 h following the injection of 3 μg TransIT-complexed U-containing mRNA, purified either by cellulose chromatography or HPLC, contained mEPO of 470 ng/mL or 370 ng/mL, respectively.

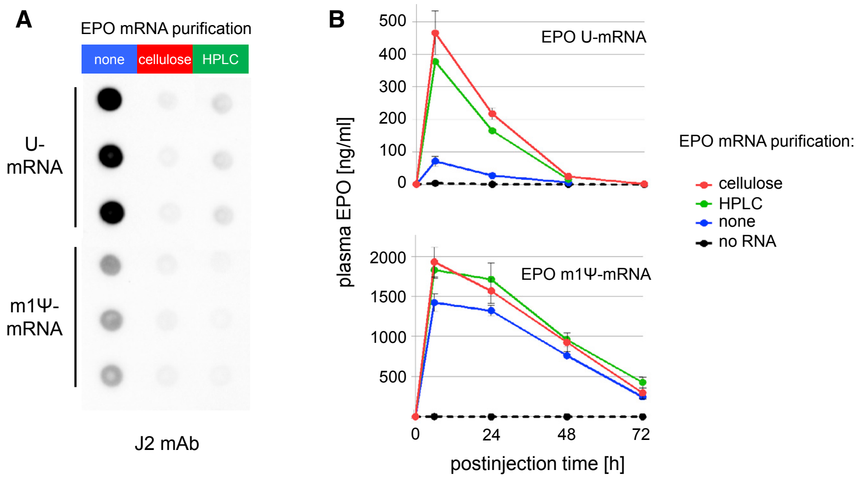

Figure 6. Translation of IVT mRNA Purified by Cellulose Chromatography

U- or m1Ψ-containing IVT mRNA encoding murine erythropoietin (mEPO) was purified either by two consecutive cycles using microcentrifuge spin columns filled with cellulose or by HPLC. (A) Unpurified, cellulose-, and HPLC-purified U- and m1Ψ-containing mEPO mRNAs (1.0 μg/dot) were analyzed in triplicates by dot blot using J2 dsRNA-specific mAb. (B) U- or m1Ψ-containing mEPO mRNAs that were unpurified and cellulose and HPLC purified were complexed with TransIT and intravenously injected into mice, and plasma mEPO levels were determined by ELISA at the indicated time. Data are represented as mean ± SD of the values obtained from 3 animals per group and are representative of three independent experiments.

Meanwhile, the corresponding unpurified U-containing mRNA led to 6.7-fold lower mEPO levels (70 ng/mL) (Figure 6B, upper panel). The impact of cellulose purification on the translatability of mRNA was even higher (7.9-fold) at 24 h after injection (Figure 6B, upper panel). The injection of unpurified, m1Ψ-containing mEPO mRNA resulted in >4-fold higher plasma mEPO levels and an extended translation kinetic compared to U-containing mEPO mRNA (Figure 6B, bottom panel). In contrast to U-containing mRNA, purification of m1Ψ-containing mRNA by cellulose chromatography and HPLC led only to a minor, statistically not significant increase in the mEPO level. This result shows that cellulose chromatography and HPLC are similarly effective in removing dsRNA contaminants from IVT mRNA and increasing its translational capacity *in vivo*.

No IFN-α Induction by Cellulose-Purified m1Ψ-Modified IVT mRNA

Removal of dsRNA contaminants from pseudouridine-containing IVT mRNA by HPLC has been reported to eliminate the secretion of interferon (IFN)-α and TNF-α by transfected dendritic cells.[17] To test the impact of cellulose purification on the IFN-α levels induced by IVT mRNA in mice, the mRNAs characterized in Figure 6 were evaluated. Mice were injected intravenously with 10 μg U- or m1Ψ-containing mRNA encoding mEPO that was complexed to form mRNA-lipoplexes, and then 6 h later IFN-α was measured in the plasma. The lipoplex formulation delivers the IVT mRNA into the spleen,[26] where plasmacytoid dendritic cells and macrophages are capable of sensitively responding to the contaminating dsRNA by secreting IFN-α.

Considering the unpurified IVT mRNAs, those that contained U induced >100-fold higher levels of IFN-α than the m1Ψ-containing mRNA (Figure 7). This immunogenic property of U-containing mRNA remained after its purification. In contrast, m1Ψ-containing mRNA that had been purified by cellulose chromatography no longer induced IFN-α.

DISCUSSION

Here we present a cellulose-based chromatography method for the elimination of dsRNA contaminants from IVT mRNAs with different lengths and nucleoside compositions. The quality and biological activity of the cellulose-purified IVT mRNA were comparable to that of the corresponding HPLC-purified mRNA. The purification method is based on the selective binding of dsRNA to cellulose powder in the presence of an ethanol-containing buffer.

The *in vitro*-synthesized RNA can be isolated from the transcription reaction by different procedures, including extraction with acidic phenol-chloroform, precipitation with LiCl, elution from silica to which RNA binds selectively in chaotropic agent, or isolation from preparative denaturing urea PAGE. In addition, chromatography methods, such as size exclusion, anion exchange, or affinity chromatography with immobilized oligo-dT, for purification of the polyadenylated IVT mRNA were also reported (reviewed by Martins et al.[27]). The aim of all these methods is to recover the RNA and eliminate proteins, nucleotides, and other components of the IVT reaction. However, removal of dsRNA contaminants by these procedures is ineffective. An early study addressed the problem of dsRNA generated during *in vitro* transcription and provided a gel purification protocol as a solution, but this method is applicable only for cleaning short RNA.[28]

The biological impacts of IVT mRNA purification have been demonstrated in different experimental systems. Decreasing viability of primary keratinocytes was reported when the cells were repeatedly exposed to unpurified rather than purified pseudouridine (Ψ)-containing IVT mRNA.[17] Reprogramming somatic cells into pluripotent stem cells with continuous treatments with Ψ-modified IVT mRNAs was significantly more potent when the transcription factor-encoding IVT mRNA was digested with RNase III, a dsRNA-specific nuclease.[29] In a recent *in vivo* experiment, improved cytotoxic killing was demonstrated by chimeric antigen receptor (CAR)-expressing T cells generated with IVT mRNA, which was digested with RNase III. In this study, T cells electroporated with either U- or m1Ψ-containing CAR mRNAs were more effective when the dsRNA was removed from the IVT mRNA.[30] This method is simple and effective, as demonstrated by the improved biological activities of the IVT mRNAs digested with RNase III.[29,30] However, incubation

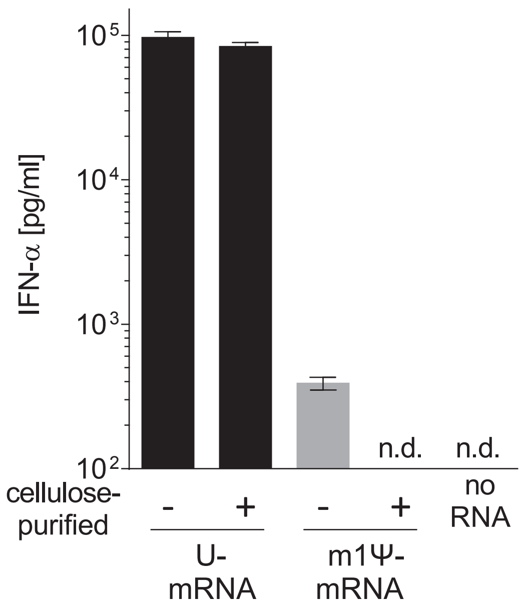

Figure 7. Induction of IFN-α by Cellulose-Purified IVT mRNA

U- or m1Ψ-containing IVT mRNA encoding mEPO was purified with cellulose in two consecutive cycles. Mice were intravenously injected with 10 μg cationic lipid-complexed unpurified or cellulose-purified mRNAs. Injection of cationic lipid containing no mRNA served as a control. Blood was collected at 6 h postinjection and serum IFN-α levels were determined by ELISA. Data are expressed as mean ± SD of the values obtained from 3 animals per group. n.d., not detectable.

with RNase III is risky, because, in addition to the dsRNA contaminants, double-stranded secondary structures formed by single-stranded RNA could be also cleaved.

Currently, the most effective way to eliminate dsRNA contaminants from long IVT mRNAs is an ion pair reversed-phase HPLC performed under semi-denaturing conditions.[17,18] Cellulose chromatography represents a new, alternative method to HPLC for the purification of IVT mRNA with several advantages. The cellulose-based method is cost effective and simple. There is no need for special equipment to perform the purification. Further, toxic acetonitrile that is used as the eluent for ion pair reversed-phase HPLC is avoided. As presented here, the cellulose-based protocol is feasible to purify IVT mRNA on the microgram-to-milligram scales, and it is also applicable for mRNAs that are as long as 4 kb. It also allows the parallel purification of up to 500 μg of several different mRNAs in less than 2 h, while HPLC purification is more time consuming due to the consecutive processing of the samples. Reusing the HPLC column also carries the risk of cross-contaminating the subsequent mRNA samples, while all material is single use in the cellulose chromatography. Finally, the rate of purified IVT mRNA recovered after cellulose chromatography is higher compared to that of HPLC. While about 50% of the input IVT mRNA is recovered after HPLC purification, using the cellulose method the recovery rate ranges between 65% and 85%, or even higher when shorter 1- to 2-kb-long mRNAs are purified. Considering that longer mRNAs are more likely to form double-stranded secondary structures, which by binding to the cellulose could result in lower recovery rates.

The isolation of dsRNA using cellulose was described decades ago,[19] but, so far, the mechanism of selective binding of dsRNA to cellulose in ethanol-containing buffer has not been elucidated. Several explanations were provided, including hydrophilic interaction between the cellulose and 2′-hydroxyl residues of dsRNA, which contains them at high density.[24] Our findings appear to be consistent with this notion, because dsDNA, DNA:RNA hybrid, and single-stranded RNA (ssRNA) that had none or a low density of 2′-hydroxyl residues did not bind to the cellulose (Figures 1A–1C). Others proposed that, during the purification, the cellulose functions as a mesh and filters the precipitated dsRNA from the soluble ssRNA.[31] However, somewhat contradicting, we found that microcrystalline cellulose that contained no tight mesh of secondary fibrils was the best cellulose type to remove dsRNA.

Composition of the chromatography buffer is a critical determinant of the cellulose selectivity for dsRNA binding. We found 125 mM NaCl and 16% (v/v) ethanol to be optimal for dsRNA removal from the IVT mRNA. When the concentration of ethanol or NaCl in the buffer was increased above the optimal values, both the dsRNA and ssRNA bound to the cellulose, while reducing their concentration had the opposite effect as less dsRNA was retained on the cellulose, leading to lower purification efficiency (data not shown).

Up until recently, the presence of dsRNA contaminant in the IVT mRNA was not recognized, as the >1-kb-long transcripts were usually analyzed for integrity by agarose gel electrophoresis, and the mRNA was assumed pure since only one band of RNA with the expected size could be visualized. Correspondingly, in the present study, we found no difference between purified and unpurified mRNA in regard to their profiles in agarose gel chromatogram (Figures 1, 4, and 5), which indicates that either the amount of dsRNA contaminant was below the limit of detection or the IVT mRNA hybridized in *cis* or *trans* to complementary RNA that was too short to noticeably alter the migration of the main mRNA product.

It has been demonstrated that HPLC removes dsRNA with heterogeneous length from IVT mRNA.[17] As we demonstrate here, cellulose efficiently binds dsRNA in the range of ≥30–1,000 bp (Figures 2 and 3), which indicates that cellulose chromatography likely removes short and long dsRNA from IVT mRNA with a similar efficiency as HPLC purification. This is further confirmed by the strong reduction of signal intensities in dot blots when cellulose- and HPLC-purified IVT mRNAs were analyzed with J2 antibody (Figure 6A), which detects ≥40-bp-long dsRNA.[25]

Considering that J2 antibody detects both m1Ψ- and U-containing dsRNAs with similar sensitivity in dot blot assays (data not shown), it seems that less dsRNA is generated during *in vitro* transcription of m1Ψ-containing IVT mRNAs as compared to the corresponding

U-containing mRNAs (Figures 4 and 6). Accordingly, Hur and colleagues,[13] while staining the IVT RNAs directly with acridine orange, also found less dsRNA by-products in the m1Ψ-RNA and more in the U-RNA samples.

The purification with either HPLC or cellulose chromatography increases the *in vivo* translatability of IVT mRNA similarly (Figure 6B), which suggests that these methods are interchangeable. Increased translation of purified IVT mRNA is likely the result of diminished activation of PKR and OAS that can be activated with dsRNA longer than 40 bp.[32]

After cellulose chromatography, m1Ψ-containing IVT mRNA does not lead to detectable levels of IFN-α *in vivo* when injected intravenously as lipoplex (Figure 7). Via this route, the IVT mRNA is delivered to the spleen where immune cells detect immunogenic RNA with high sensitivity.[26] This demonstrates the removal of IFN-inducing dsRNA from m1Ψ-containing IVT mRNA with cellulose purification. In contrast, U-containing IVT mRNA induces a strong IFN-α response, even after cellulose purification. This is in line with published results showing that HPLC-purified U-containing mRNA leads to high IFN-α in dendritic cells.[17] This response, however, is not dsRNA dependent but due to the activation of TLR7 by single-stranded U-containing mRNA.[33]

Conclusively, the cellulose chromatography described here represents a cost-effective and simple method to remove dsRNA contaminants, resulting in pure IVT mRNA of a quality similar to HPLC-purified mRNA.

MATERIALS AND METHODS

IVT mRNA Synthesis

mRNAs were transcribed from linearized plasmids encoding firefly Luc, codon-optimized mEPO, EGFP, or hiNOS using T7 RNA polymerase (MEGAscript T7 Transcription Kit, Thermo Fisher Scientific, Darmstadt, Germany). The mRNAs were transcribed to contain the 5′ UTR derived from the tobacco etch virus 5′ leader RNA (TEV).[34] Further, a 100-nt-long poly(A) tail interrupted by a short linker (A30LA70) was transcribed from the corresponding DNA templates. For the generation of nucleoside-modified mRNAs, uridine 5’-triphosphate (UTP) was replaced with the triphosphate derivative of m1Ψ (m1ΨTP) in the transcription reaction. Capping of the IVT mRNAs was performed co-transcriptionally using the trinucleotide cap1 analog CleanCap (TriLink, San Diego, CA, USA). After DNase digestion, the synthesized mRNA was isolated from the reaction mix by precipitation with half volume of 8 M LiCl solution (Sigma-Aldrich, Hamburg, Germany), and finally the pellet was dissolved in nuclease-free water. The RNA concentration was determined using a Nanodrop 2000c spectrophotometer (Thermo Fisher Scientific). Aliquots of denatured IVT mRNAs were analyzed by electrophoresis in non-denaturing 1.4% agarose gels containing 0.005% (v/v) GelRed nucleic acid gel stain.[35] RiboRuler High Range RNA Ladder (Thermo Fisher Scientific) was loaded as a molecular weight marker.

Generation of dsRNA

Plasmids containing a 1-kb-long insert in either direct or reverse orientations downstream of the promoter of T7 RNA polymerase were used as templates. Complementary m1Ψ- or U-containing RNAs were *in vitro* transcribed and purified by cellulose chromatography to remove IVT-derived dsRNA contaminants. Annealing of the complementary strands was performed by heating to 95°C for 1 min in buffer (10 mM Tris-HCl [pH 7.0] and 50 mM NaCl) and cooling to room temperature over a period of 2 h. After precipitation with isopropanol, the dsRNA pellet was dissolved in nuclease-free water, and the remaining ssRNA was eliminated by digestion with 1 unit S1 nuclease (Thermo Fisher Scientific) /μg dsRNA at room temperature for 45 min. Finally, the dsRNA free of ssRNA was isolated by phenol-chloroform extraction, recovered by isopropanol precipitation, and dissolved in buffer (0.1 mM EDTA and 10 mM HEPES [pH 7.0]).

Cellulose-Based Purification of IVT mRNA

Removal of dsRNA contaminants from 100 to 500 μg IVT mRNA was performed using microcentrifuge spin columns (NucleoSpin Filters, Macherey-Nagel, Düren, Germany), cellulose fibers (C6288, Sigma-Aldrich), and a chromatography buffer containing 10 mM HEPES (pH 7.2), 0.1 mM EDTA, 125 mM NaCl, and 16% (v/v) ethanol. The cellulose was first prewashed, by suspending in chromatography buffer at a concentration of 0.2 g cellulose/mL and incubated for 10 min under vigorous shaking. Next, 700 μL cellulose slurry (0.14 g cellulose) was transferred to a microcentrifuge spin column and centrifuged for 60 s at 14,000 × *g*. The flowthrough was discarded, and 500 μL chromatography buffer was added to the spin column and shaken vigorously for 5 min to resuspend the cellulose within the spin column. After centrifugation for 60 s at 14,000 × *g*, the flowthrough was discarded and 100–500 μg IVT mRNA in 500 μL chromatography buffer was added to the spin column containing the prewashed cellulose. During the subsequent 30 min, the IVT mRNA and cellulose slurry were shaken vigorously at room temperature to promote resuspension of the cellulose and its association with the dsRNA contaminant. By centrifugation of the spin column for 60 s at 14,000 × *g*, the cellulose together with the associated dsRNA contaminant was separated from the unbound single-stranded IVT mRNA in the flowthrough. Where indicated, the unbound fraction containing the single-stranded IVT mRNA was directly transferred to a second spin column containing prewashed cellulose, and the 30-min incubation procedure was repeated (2 cycles of purification). Finally, the purified mRNA was recovered from the unbound fraction by adding 0.1 vol 3 M NaOAc (pH 5.5) and 1 vol isopropanol. The precipitated RNA was collected by centrifugation at 14,000 × *g* and dissolved in nuclease-free water. Sometimes, the unbound fraction contained a tiny amount of particulate cellulose material that was visible after centrifugation as a white pellet. Cellulose particles were avoided using microcentrifuge spin columns containing a 0.2- or 0.45-μm filter (e.g., Ultrafiltration Spin-Columns, 0.45 μm cutoff, Merck, Darmstadt, Germany).

In some experiments, 6-kb linearized plasmid and 1-kb U-containing dsRNA were mixed and subjected to cellulose-based purification.

Plasmid DNA and dsRNA were recovered from the unbound and the cellulose-bound fractions, respectively, and loaded to non-denaturing 1% agarose gels containing 0.005% (v/v) GelRed nucleic acid gel stain. GeneRuler 1 kb DNA Ladder (Thermo Fisher Scientific) was loaded as a molecular weight marker. To determine the dsRNA-binding capacity of the cellulose, one cycle of cellulose-chromatography of 0.25–25 μg dsRNA using a spin column filled with 0.14 g cellulose was performed. The presence of dsRNA in the unbound fraction indicated the over-saturation of the cellulose.

For FPLC-based cellulose chromatography of 100 mg IVT mRNA, a suspension of 250 g cellulose in 1,400 mL chromatography buffer (0.18 g/mL) was prepared in a beaker by manually stirring to generate homogeneous slurry. The slurry was then transferred to an XK 50/60 chromatography column (GE Healthcare Life Sciences, Freiburg, Germany), and the outlet of the column was opened, allowing the cellulose bed to settle by gravity. The cellulose-filled column was then connected to Äkta avant 25 FPLC system and washed with 780 mL chromatography buffer (1-column volume) at a flow rate of 5 mL/min. Then 100 mL mRNA sample in chromatography buffer (1 mg/mL mRNA final concentration) was loaded to the column, and the chromatography was performed at a flow rate of 5 mL/min. The UV absorbance of the flowthrough was monitored at 260 nm, and the peak fraction corresponding to the eluted IVT mRNA was collected. Later in the run, the chromatography buffer was changed to nuclease-free water to release the cellulose-bound nucleic acid containing the dsRNA contaminants. For further analysis, the nucleic acid from the collected chromatographic peaks was recovered by adding 0.1 vol 3 M NaOAc (pH 5.5) and 1 vol isopropanol, followed by precipitation.

Cellulose-Based Purification and Gel Electrophoresis of dsRNA Ladder

The total of 2.5 μg broad-range dsRNA ladder (21–500 bp, New England Biolabs, Frankfurt, Germany) was purified by cellulose chromatography. After 10- or 30-min incubation with cellulose, 2 μL of the unbound and cellulose-bound fractions was loaded onto a non-denaturing 2.2% agarose gel and a native 21% polyacrylamide gel. To visualize the dsRNA, the agarose gel contained 0.005% (v/v) GelRed, while the polyacrylamide gel was stained post-run with SYBR Gold (Thermo Fisher Scientific) diluted 1:10,000 in Tris/acetic acid/EDTA (TAE) buffer. Images were taken using Bio-Rad EZ Gel Documentation System.

HPLC Purification of IVT mRNA

RNA was purified by HPLC using a Semi-Prep RNASep (100 × 21.1-mm) column packed with a matrix of C-18 alkylated non-porous polystyrene-divinylbenzene copolymer microspheres (Transgenomics, Omaha, NE) connected to an Äkta avant 25 system. Ion pair reversed-phase chromatography was performed according to a protocol described previously,[17,18] using a linear gradient of 38%–70% buffer B (0.1 M triethylammonium acetate [TEAA, pH 7.0] and 25% [v/v] acetonitrile) in buffer A (0.1 M TEAA [pH 7.0]) at a flow rate of 5 mL/min. The mRNA from the collected peak fractions was concentrated and desalted, by successive centrifugation using Amicon Ultra-15 centrifugal filter units (30 kDa molecular weight cut-off) (Merck Millipore, Darmstadt, Germany) and dilution with nuclease-free water, and finally it was recovered from the retentate by isopropanol precipitation.

Detection of dsRNA and RNA:DNA Hybrids by Dot Blot Analysis

The IVT mRNA samples were diluted in nuclease-free water to final concentrations of 8, 40, 200, and 600 ng/μL, and 5-μL aliquots were then spotted to a positively charged nylon membrane (Whatman Nytran SuPerCharge, Sigma-Aldrich), resulting in a total amount of 40, 200, 1,000, and 3,000 ng IVT mRNA per dot, respectively. Before loading, the membrane was placed on a sheet of Whatman GB005 blotting paper and fixed with tape. Then a silicone mask (Bio-Dot Gasket 96 wells, Bio-Rad, Munich, Germany) was tightly pressed onto the membrane to ensure that all wells were sealed to avoid sample leakage. Loading was performed by pipetting 5 μL diluted sample into the wells of the silicone mask. This was achieved by placing the pipette tip vertically over the center of the well to ensure equal sample distribution on the area defined by the well. The sample liquid drained into the membrane and the underlying blotting paper by capillary forces, while the negatively charged RNA was captured by the positively charged nylon membrane. After loading, the sealing gasket was removed and the air-dried membrane transferred to a 50-mL tube and blocked in 5% (w/v) non-fat dried milk in Tris-buffered saline (TBS)-T buffer (20 mM Tris [pH 7.4], 150 mM NaCl, and 0.1% (v/v) Tween-20).

For the detection of dsRNA, the membrane was incubated with J2 anti-dsRNA murine antibody (Scicons, Budapest, Hungary) diluted 1:5,000. To detect RNA:DNA hybrids, the incubation was performed with S9.6 murine monoclonal antibody (mAb) (Kerafast, Boston, MA, USA) diluted 1:20,000. The antibodies were diluted in 1% (w/v) non-fat dried milk and TBS-T and incubated with the membranes on a rolling mixer at 4°C overnight. The membranes were washed three times with 35 mL TBS-T for 15 min each and then incubated with horseradish peroxidase (HRP)-conjugated donkey anti-mouse immunoglobulin G (IgG) (Jackson ImmunoResearch Laboratories, Cambridgeshire, UK) diluted 1:10,000 in 1% (w/v) non-fat dried milk and TBS-T at room temperature for 1 h. After washing the membranes three times with 35 mL TBS-T for 15 min, chemiluminescence detection was performed using Amersham ECL Prime Western Blotting Detection Reagent (GE Healthcare Life Sciences) and the ChemiDoc MP Imaging System (Bio-Rad). The signal intensities of the dots were quantified by densitometry using the Volume Tools of the Image Lab software (Bio-Rad). To verify equal sample loading, membranes were stripped with 1% (w/v) SDS containing 40 mM dithiothreitol at 60°C for 30 min and probed with a 3′-biotinylated oligodeoxynucleotide (5′-GTG AGT GGG GCA GGT GGA GGT GGG AGC ATA-3′) complementary to the 3′ UTR of the mRNA. Reprobing with oligodeoxynucleotide (ODN) was performed in PerfectHyb Plus Hybridization Buffer (Sigma-Aldrich), according to the manufacturer, and HRP-conjugated streptavidin (Thermo Fisher Scientific) was used for visualization.

Complexing of mRNA

RNA was complexed with TransIT-mRNA (Mirus Bio, Madison, WI, USA) according to the manufacturer. A ratio of mRNA (3 μg), TransIT-mRNA reagent (3.3 μL), and boost reagent (2.1 μL) in a final volume of 200 μL DMEM was used. For complexing different amounts of mRNA, the volumes of the reagents and the final volume were scaled proportionally. For *in vivo* delivery to lymphoid compartments, mRNA was complexed with cationic lipid to form RNA-lipoplexes, as described by Kranz et al.[26]

Animal Experiments

All experiments were performed in accordance with federal policies on animal research using BALB/c female mice from Charles River Laboratories (Sandhofer, Germany) at an age of 6–12 weeks. For determining the translation of mRNA *in vivo*, 3 μg cap1-TEV-mEPO mRNA complexed with TransIT was injected intravenously into mice (3 mice/group). Blood was collected at 6, 24, 48, and 72 h after mRNA injection as described[36,37] to avoid an impact of the sampling on the hematological parameters of the animals. In brief, blood (18 μL) was collected by puncture of the tail vein, mixed with 2 μL 0.2 M EDTA, and centrifuged in 20 μL Drummond microcaps glass microcapillary tubes (Sigma-Aldrich). After snapping the microcapillary tubes, the plasma was recovered for the measurement of plasma mEPO levels using the mEPO DuoSet ELISA Development kit (R&D Systems, Minneapolis, MN, USA) and the Infinite 200 Pro plate reader (Tecan, Mannedorf, Switzerland).

To determine if IFN-α is induced by mRNA *in vivo* in mice, 10 μg cap1-TEV-mEPO mRNA was injected intravenously as RNA-lipoplexes in a final volume of 200 μL (3 mice/group). The animals were sacrificed 6 h after injection, and blood was collected, transferred to Serum-Gel Z tubes (Sarstedt, Numbrecht, Germany), and centrifuged at 2,500 × *g* for 10 min to obtain serum. IFN-α levels in the serum were determined using a murine IFN-α ELISA kit (BMS6027, Thermo Fisher Scientific).

SUPPLEMENTAL INFORMATION

Supplemental Information can be found online at https://doi.org/10.1016/j.omtn.2019.02.018.

AUTHOR CONTRIBUTIONS

M.B., U.S., and K.K. designed the experiments. M.B., G.B., H.M., A.M., and I.V. conducted the experiments. M.B. and K.K. wrote the paper.

CONFLICTS OF INTEREST

All authors are employees of BioNTech RNA Pharmaceuticals, a company that develops mRNA-based therapies. M.B. and K.K. have a pending patent application titled “Methods for Providing Single-Stranded RNA” for the technology presented in this paper.

ACKNOWLEDGMENTS

Funding for the experiments was provided by BioNTech RNA Pharmaceuticals.

REFERENCES

1. Sahin, U., Karikó, K., and Türeci, Ö. (2014). mRNA-based therapeutics–developing a new class of drugs. Nat. Rev. Drug Discov. *13*, 759–780.
2. Sultana, S., Solotchi, M., Ramachandran, A., and Patel, S.S. (2017). Transcriptional fidelities of human mitochondrial POLRMT, yeast mitochondrial Rpo41, and phage T7 single-subunit RNA polymerases. J. Biol. Chem. *292*, 18145–18160.
3. Milligan, J.F., Groebe, D.R., Witherell, G.W., and Uhlenbeck, O.C. (1987). Oligoribonucleotide synthesis using T7 RNA polymerase and synthetic DNA templates. Nucleic Acids Res. *15*, 8783–8798.
4. Martin, C.T., Muller, D.K., and Coleman, J.E. (1988). Processivity in early stages of transcription by T7 RNA polymerase. Biochemistry *27*, 3966–3974.
5. Konarska, M.M., and Sharp, P.A. (1989). Replication of RNA by the DNA-dependent RNA polymerase of phage T7. Cell *57*, 423–431.
6. Krupp, G. (1989). Unusual promoter-independent transcription reactions with bacteriophage RNA polymerases. Nucleic Acids Res. *17*, 3023–3036.
7. Cazenave, C., and Uhlenbeck, O.C. (1994). RNA template-directed RNA synthesis by T7 RNA polymerase. Proc. Natl. Acad. Sci. USA *91*, 6972–6976.
8. Triana-Alonso, F.J., Dabrowski, M., Wadzack, J., and Nierhaus, K.H. (1995). Self-coded 3′-extension of run-off transcripts produces aberrant products during in vitro transcription with T7 RNA polymerase. J. Biol. Chem. *270*, 6298–6307.
9. Biebricher, C.K., and Luce, R. (1996). Template-free generation of RNA species that replicate with bacteriophage T7 RNA polymerase. EMBO J. *15*, 3458–3465.
10. Arnaud-Barbe, N., Cheynet-Sauvion, V., Oriol, G., Mandrand, B., and Mallet, F. (1998). Transcription of RNA templates by T7 RNA polymerase. Nucleic Acids Res. *26*, 3550–3554.
11. Nacheva, G.A., and Berzal-Herranz, A. (2003). Preventing nondesired RNA-primed RNA extension catalyzed by T7 RNA polymerase. Eur. J. Biochem. *270*, 1458–1465.
12. Gholamalipour, Y., Karunanayake Mudiyanselage, A., and Martin, C.T. (2018). 3′ end additions by T7 RNA polymerase are RNA self-templated, distributive and diverse in character-RNA-Seq analyses. Nucleic Acids Res. *46*, 9253–9263.
13. Mu, X., Greenwald, E., Ahmad, S., and Hur, S. (2018). An origin of the immunogenicity of in vitro transcribed RNA. Nucleic Acids Res. *46*, 5239–5249.
14. Katayama, S., Tomaru, Y., Kasukawa, T., Waki, K., Nakanishi, M., Nakamura, M., Nishida, H., Yap, C.C., Suzuki, M., Kawai, J., et al.; RIKEN Genome Exploration Research Group; Genome Science Group (Genome Network Project Core Group); FANTOM Consortium (2005). Antisense transcription in the mammalian transcriptome. Science *309*, 1564–1566.
15. Portal, M.M., Pavet, V., Erb, C., and Gronemeyer, H. (2015). Human cells contain natural double-stranded RNAs with potential regulatory functions. Nat. Struct. Mol. Biol. *22*, 89–97.
16. Hartmann, G. (2017). Nucleic Acid Immunity. Adv. Immunol. *133*, 121–169.
17. Karikó, K., Muramatsu, H., Ludwig, J., and Weissman, D. (2011). Generating the optimal mRNA for therapy: HPLC purification eliminates immune activation and improves translation of nucleoside-modified, protein-encoding mRNA. Nucleic Acids Res. *39*, e142.
18. Weissman, D., Pardi, N., Muramatsu, H., and Karikó, K. (2013). HPLC purification of in vitro transcribed long RNA. Methods Mol. Biol. *969*, 43–54.
19. Franklin, R.M. (1966). Purification and properties of the replicative intermediate of the RNA bacteriophage R17. Proc. Natl. Acad. Sci. USA *55*, 1504–1511.
20. Day, P.R., Dodds, J.A., Elliston, J.E., Jaynes, S.L., and Anagnostakis, S.L. (1977). Double-stranded-RNA in Endothia parasitica. Phytopathology *67*, 1393–1396.
21. Morris, T.J., and Dodds, J.A. (1979). Isolation and Analysis of Double-Stranded RNA from Virus-Infected Plant and Fungal Tissue. Phytopathology *69*, 854–858.
22. Castillo, A., Cottet, L., Castro, M., and Sepúlveda, F. (2011). Rapid isolation of mycoviral double-stranded RNA from Botrytis cinerea and Saccharomyces cerevisiae. Virol. J. *8*, 38.
23. Okada, R., Kiyota, E., Moriyama, H., Toshiyuki, F., and Valverde, R.A. (2014). A new endornavirus species infecting Malabar spinach (Basella alba L.). Arch. Virol. *159*, 807–809.

24. Urayama, S., Yoshida-Takashima, Y., Yoshida, M., Tomaru, Y., Moriyama, H., Takai, K., and Nunoura, T. (2015). A New Fractionation and Recovery Method of Viral Genomes Based on Nucleic Acid Composition and Structure Using Tandem Column Chromatography. Microbes Environ. *30*, 199–203.

25. Bonin, M., Oberstrass, J., Lukacs, N., Ewert, K., Oesterschulze, E., Kassing, R., and Nellen, W. (2000). Determination of preferential binding sites for anti-dsRNA antibodies on double-stranded RNA by scanning force microscopy. RNA *6*, 563–570.

26. Kranz, L.M., Diken, M., Haas, H., Kreiter, S., Loquai, C., Reuter, K.C., Meng, M., Fritz, D., Vascotto, F., Hefesha, H., et al. (2016). Systemic RNA delivery to dendritic cells exploits antiviral defence for cancer immunotherapy. Nature *534*, 396–401.

27. Martins, R., Queiroz, J.A., and Sousa, F. (2014). Ribonucleic acid purification. J. Chromatogr. A *1355*, 1–14.

28. Mellits, K.H., Pe'ery, T., Manche, L., Robertson, H.D., and Mathews, M.B. (1990). Removal of double-stranded contaminants from RNA transcripts: synthesis of adenovirus VA RNAI from a T7 vector. Nucleic Acids Res. *18*, 5401–5406.

29. Meis, J., Person, A., Chin, C., Jendrisak, J., and Dahl, G. (2016). Making and using in vitro-synthesized ssRNA for introducing into mammalian cells to induce a biological or biochemical effect. EU patent 2,798,064, filed December 31, 2012, and granted August 31, 2016.

30. Foster, J.B., Choudhari, N., Perazzelli, J., Storm, J., Hofmann, T.J., Jain, P., Storm, P.B., Pardi, N., Weissman, D., Waanders, A.J., et al. (2019). Purification of mRNA Encoding Chimeric Antigen Receptor Is Critical for Generation of a Robust T-Cell Response. Hum. Gene Ther. *30*, 168–178.

31. Su, X., and Comeau, A.M. (1999). Cellulose as a matrix for nucleic acid purification. Anal. Biochem. *267*, 415–418.

32. Minks, M.A., West, D.K., Benvin, S., and Baglioni, C. (1979). Structural requirements of double-stranded RNA for the activation of 2′,5′-oligo(A) polymerase and protein kinase of interferon-treated HeLa cells. J. Biol. Chem. *254*, 10180–10183.

33. Diebold, S.S., Kaisho, T., Hemmi, H., Akira, S., and Reis e Sousa, C. (2004). Innate antiviral responses by means of TLR7-mediated recognition of single-stranded RNA. Science *303*, 1529–1531.

34. Gallie, D.R., Tanguay, R.L., and Leathers, V. (1995). The tobacco etch viral 5′ leader and poly(A) tail are functionally synergistic regulators of translation. Gene *165*, 233–238.

35. Masek, T., Vopalensky, V., Suchomelova, P., and Pospisek, M. (2005). Denaturing RNA electrophoresis in TAE agarose gels. Anal. Biochem. *336*, 46–50.

36. Karikó, K., Muramatsu, H., Keller, J.M., and Weissman, D. (2012). Increased erythropoiesis in mice injected with submicrogram quantities of pseudouridine-containing mRNA encoding erythropoietin. Mol. Ther. *20*, 948–953.

37. Mahiny, A.J., and Karikó, K. (2016). Measuring Hematocrit in Mice Injected with In Vitro-Transcribed Erythropoietin mRNA. Methods Mol. Biol. *1428*, 297–306.

OMTN, Volume *15*

Supplemental Information

A Facile Method for the Removal of dsRNA Contaminant from *In Vitro*-Transcribed mRNA

Markus Baiersdörfer, Gábor Boros, Hiromi Muramatsu, Azita Mahiny, Irena Vlatkovic, Ugur Sahin, and Katalin Karikó

Supplementary Fig. 1

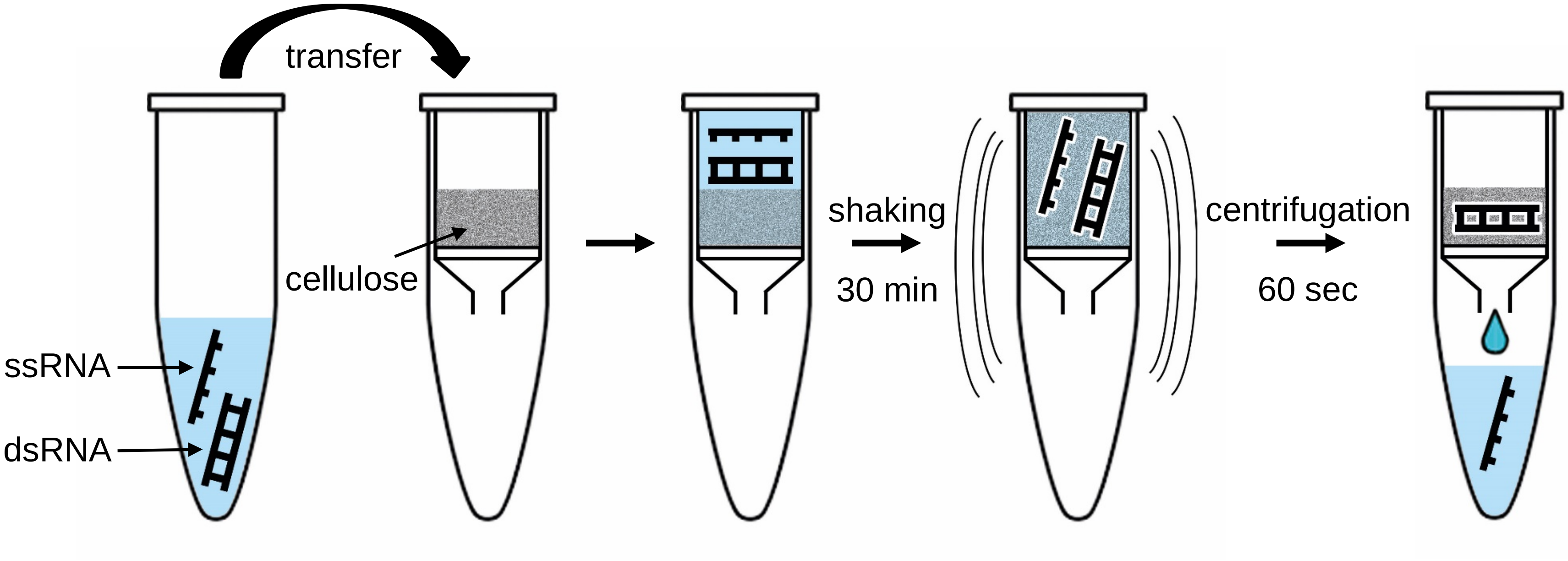

Supplementary Fig. 2

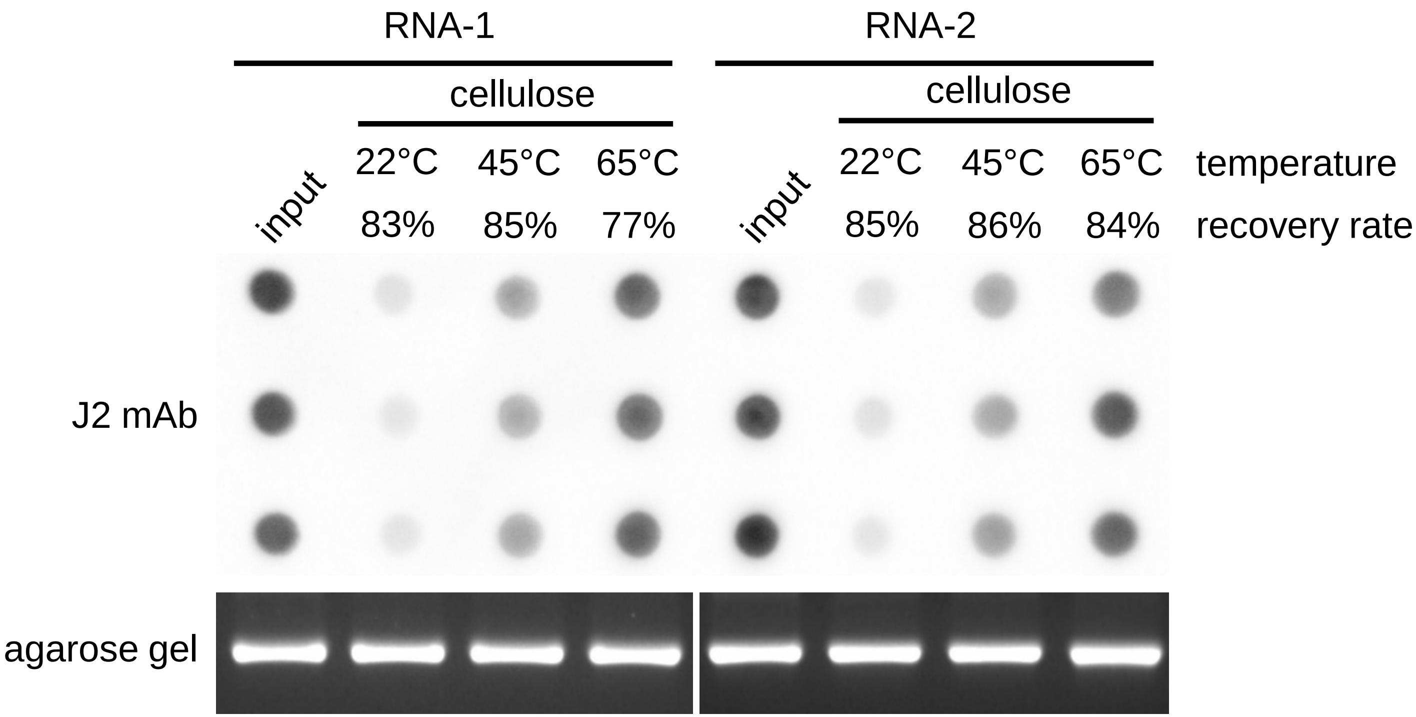

SUPPLEMENTARY MATERIALS

Supplementary Figure 1. Schematic overview of the cellulose-based purification method using microcentrifuge spin column. IVT mRNA in chromatography buffer containing 16% (v/v) ethanol is transferred to cellulose-loaded microcentrifuge spin column. The spin column is shaken vigorously at room temperature for 30 minutes to promote resuspension of the cellulose and its association with the dsRNA contaminant contained in the IVT mRNA. By centrifugation of the spin column at 14,000 x g for 60 seconds the cellulose together with the associated dsRNA contaminant is separated from the unbound single-stranded IVT mRNA (ssRNA) in the flow through.

Supplementary Figure 2. High temperatures decrease the efficiency of dsRNA removal with cellulose chromatography. Two different uridine-containing, 1.9 kb-long IVT mRNAs (RNA-1 and RNA-2) were purified at 22°C, 45°C or 65°C using cellulose-filled microcentrifuge spin columns. Aliquots of the unpurified (input) and cellulose-purified IVT mRNAs (1 µg per dot) were analyzed in triplicates on dot blot with J2 dsRNA-specific mAb for presence of dsRNA contaminants. The recovery rates of the purified mRNAs relative to the input mRNA are noted. The integrities of unpurified and cellulose-purified IVT mRNAs were analyzed by electrophoresis on a 1.4% agarose gel. The GelRed-stained RNAs were visualized by UV illumination.
